# Quality of radiotherapy reporting in randomized controlled trials of prostate cancer

**DOI:** 10.1186/s13014-018-1053-7

**Published:** 2018-06-07

**Authors:** Yu Yang Soon, Desiree Chen, Teng Hwee Tan, Jeremy Tey

**Affiliations:** Department of Radiation Oncology, National Cancer Institue of Singapore, National University Hospital, 5 Lower Kent Road, Singapore, 119074 Singapore

**Keywords:** Quality of radiotherapy reporting, Prostate cancer, Randomized controlled trials

## Abstract

**Background:**

Good radiotherapy reporting in clinical trials of prostate radiotherapy is important because it will allow accurate reproducibility of radiotherapy treatment and minimize treatment variations that can affect patient outcomes. The aim of our study is to assess the quality of prostate radiotherapy (RT) treatment reporting in randomized controlled trials in prostate cancer.

**Methods:**

We searched MEDLINE for randomized trials of prostate cancer, published from 1996 to 2016 and included prostate RT as one of the intervention arms. We assessed if the investigators reported the ten criteria adequately in the trial reports: RT dose prescription method; RT dose-planning procedures; organs at risk (OAR) dose constraints; target volume definition, simulation procedures; treatment verification procedures; total RT dose; fractionation schedule; conduct of quality assurance (QA) as well as presence or absence of deviations in RT treatment planning and delivery. We performed multivariate logistic regression to determine the factors that may influence the quality of reporting.

**Results:**

We found 59 eligible trials. There was significant variability in the quality of reporting. Target volume definition, total RT dose and fractionation schedule were reported adequately in 97% of included trials. OAR constraints, simulation procedures and presence or absence of deviations in RT treatment planning and delivery were reported adequately in 30% of included trials. Twenty-four trials (40%) reported seven criteria or more adequately. Multivariable logistic analysis showed that trials that published their quality assurance results and cooperative group trials were more likely to have adequate quality in reporting in at least seven criteria.

**Conclusion:**

There is significant variability in the quality of reporting on prostate radiotherapy treatment in randomized trials of prostate cancer. We need to have consensus guidelines to standardize the reporting of radiotherapy treatment in randomized trials.

**Electronic supplementary material:**

The online version of this article (10.1186/s13014-018-1053-7) contains supplementary material, which is available to authorized users.

## Background

The results from randomized clinical trials (RCTs) provides the best clinical evidence and forms that basis for clinical practice in medicine [1]. The knowledge gleaned from RCTs can change the clinical management of patients and practice patterns of clinicians. Given the importance of RCTs, sufficient information on intervention details must be provided in the trial reports to allow the reader to come to his or her conclusions about the recommended treatment. There must be a minimum standard in the quality of reporting of randomized trials so that information is presented in a complete and unambiguous manner [2]. For radiation oncology, the quality of radiotherapy (RT) reporting is important for several reasons. Firstly, radiation treatment parameters such as the radiation prescription, planning process and treatment delivery should be clearly reported to allow accurate reproducibility of the treatment in real world practice. Secondly, clear specifications of treatment parameters will minimize treatment variations that can affect patient outcomes. Thirdly, radiation oncology is a discipline, which is heavily dependent on quality assurance (QA) [3]. Newer and modern developments in radiation oncology have led to new planning systems and treatment delivery machines being used for patient treatment. Routine reporting of the use of QA is now expected in RCTs to ensure that participating centers are able to deliver radiation in a clinically consistent and reproducible manner.

The CONSORT statement provides evidence based minimum set of recommendations for reporting of randomized trials [4]. It aims to help authors prepare their reports in a transparent and unbiased manner. The details of the ‘interventions’ in the checklist of the CONSORT checklist entails reporting of the interventions for each group with sufficient details to allow replication of the intervention [5]. However, the minimum standard of reporting for intervention will vary from one discipline to the other. In addition, there is limited evidence available on the reporting of radiotherapy treatment in RCTs.

Prostate cancer has the highest incidence amongst men in developed countries [6]. External beam radiotherapy (EBRT) is one of the options in the curative treatment of prostate cancer. Many RCTs have been conducted to improve the outcome of patients with prostate cancer, both in terms of improving biochemical disease free survival, overall survival and reducing treatment toxicity. Radiation treatment has advanced from 2 dimensional radiotherapy (2DRT), to 3 dimensional conformal radiotherapy (3DCRT) and to advanced technologies such as intensity modulated radiotherapy (IMRT) and proton beam therapy in the modern era today [7].

A previous report by Bekelman and colleagues examined the quality of radiotherapy reporting in RCTs of Hodgkin’s and Non-Hodgkin’s lymphoma and showed that the reporting of radiotherapy was deficient [8]. Only 38% of the included trials described the target volume; 21% of trials specified the RT prescription point and 20% of included trials described using a RT QA process. It is unclear if the same observation can be made for RCTs of prostate cancer.

The aim of our study is to assess the quality of prostate RT treatment reporting in RCTs of prostate cancer, the factors associated with adequate quality reporting and its impact on the bias in reporting of the trial’s primary efficacy and toxicity outcomes.

## Methods

### Trial criteria

This study incorporated randomized trials including patients with histologically proven prostate cancer. At least one of the intervention arms needed to include curative intent radiotherapy treatment to the prostate. If the trial had more than one report, all the reports were assessed but would be counted as one trial. We used the trial protocol in the assessment of quality of radiotherapy reporting if they were referenced in the trial report or provided as supplementary materials accompanying the trial report.

### Search strategy

From January 1996 to December 2016, we searched MEDLINE (PubMed) for “Prostate neoplasms” and “Radiotherapy”. We limited our search to publications in the English language and to randomized trials. We then hand searched the results for eligible trials. The final selection was made from reading the full text article.

### Data extraction

We had 3 independent reviewers using a standardized form to collect the trial data.. We extracted trial characteristics such as sample size, interventions, primary outcome measures and publication characteristics such as year of publication, impact factor of journal in the year of publication for each report.

### Quality assessment

We assessed the quality of RT treatment reporting in the trial report primarily and used information in the trial protocol for assessment if it was mentioned in the trial report or was part of supplementary materials. Based on the Radiation Therapy Oncology Group (RTOG) Procedure Manual (available from https://www.rtog.org/Researchers/PoliciesManuals/ProceduresManual.aspx), we used 10 RT reporting measures to assess the quality of the prostate RT treatment reporting. The 10 measures are: RT dose prescription method; RT dose-planning procedures; organs at risk (OAR) dose constraints; target volume definition, simulation procedures; treatment verification procedures; total RT dose; fractionation schedule; conduct of QA as well as presence or absence of deviations in RT treatment planning and delivery. The adequacy definitions for these measures are summarized in Table 1. Any differences were resolved consensually between the 3 reviewers. For each trial, key descriptors were scored as “No” if they were absent in the trial report/ protocol, and “yes” if these were reported We defined a trial as having adequate quality in reporting of RT treatment if seven or more key descriptors were reported adequately.

**Table 1:** The Key Descriptors (quality measures) and the corresponding definition for adequacy. Ten Key Descriptors are described: Target volume definition, definition of dose planning procedures, radiation dose specification, fractionation specification, radiation prescription point specification, OAR constraints, simulation procedures, verification procedures, QA process for RT, QA process adherence reporting for RT

Key descriptors	Adequacy definition
Target volume definition	Define the Clinical Target Volume at least
Description of dose planning procedure	Must describe if inverse/forward planned, static IMRT vs dynamic/arc therapy
Radiation dose specification	Describe total dose and dose per fraction in centigray, Gray or rads
Fractionation specification	Describe the number and timing of fractions administered
Radiation prescription point specification	Describe the depth of radiation prescription point (for 3D conformal RT technique) or volume based dose prescription (for intensity modulated or Arc RT technique)
OAR constraints	Report OAR constraints
Simulation procedures	Report setup position or any rectal and bowel preparation
Verification procedures	Report the use of any verification procedures such as cone beam CT, EPI (electronic portal imaging), tracking
QA process for RT	Report the use of QA process for RT delivery
QA process adherence reporting for RT	Define deviations from protocol in RT delivery

### Assessment of bias in the reporting of the trial’s primary efficacy and toxicity outcomes

**Fig. 1:**
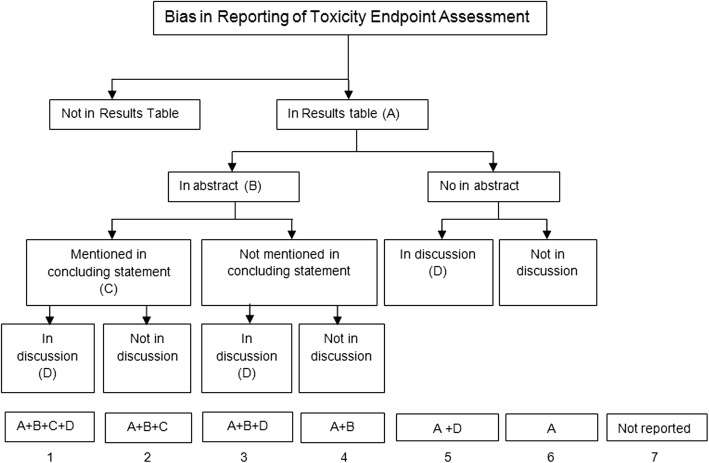
Bias in reporting of toxicity endpoint assessment was explored using a hierarchy scale from 1 (excellent) to 7 (very poor) to indicate whether reporting of toxicities occurred in the abstract, discussion, concluding statement or results table (Fig. 1). Reports with scores 5 to 7 were judged as having bias in the reporting of toxicity endpoint

We defined biased reporting using published criteria [9]. Bias in the reporting of primary efficacy endpoint is defined as no statistical significant difference in primary efficacy endpoint yet one of the treatment arms was highlighted as beneficial. Bias in the reporting of toxicity endpoint was explored using a hierarchy scale from 1 (excellent) to 7 (very poor) to indicate whether reporting of toxicities occurred in the abstract, discussion, concluding statement or results table (Fig. 1). Reports with scores 5 to 7 were judged as having bias in the reporting of toxicity endpoint.

### Statistical analysis

The descriptive statistics were presented as frequencies and percentages. Multivariable logistic regression was performed (using forward selection) to determine the potential factors associated with adequate quality reporting. Continuous variables such as impact factor, year of publication and sample size were recategorized as dichotomized nominal variables into various categories determined a priori. Univariable logistic regression was used to determine the impact of adequate reporting on the bias of reporting of primary efficacy and toxicity endpoints. Factors with *P* < 0.05 in logistic regression were considered statistically significant. All statistical analyses were performed using Stata version 13 (StataCorp LP, TX, USA).

## Results

### Selection of trials

**Fig. 2:**
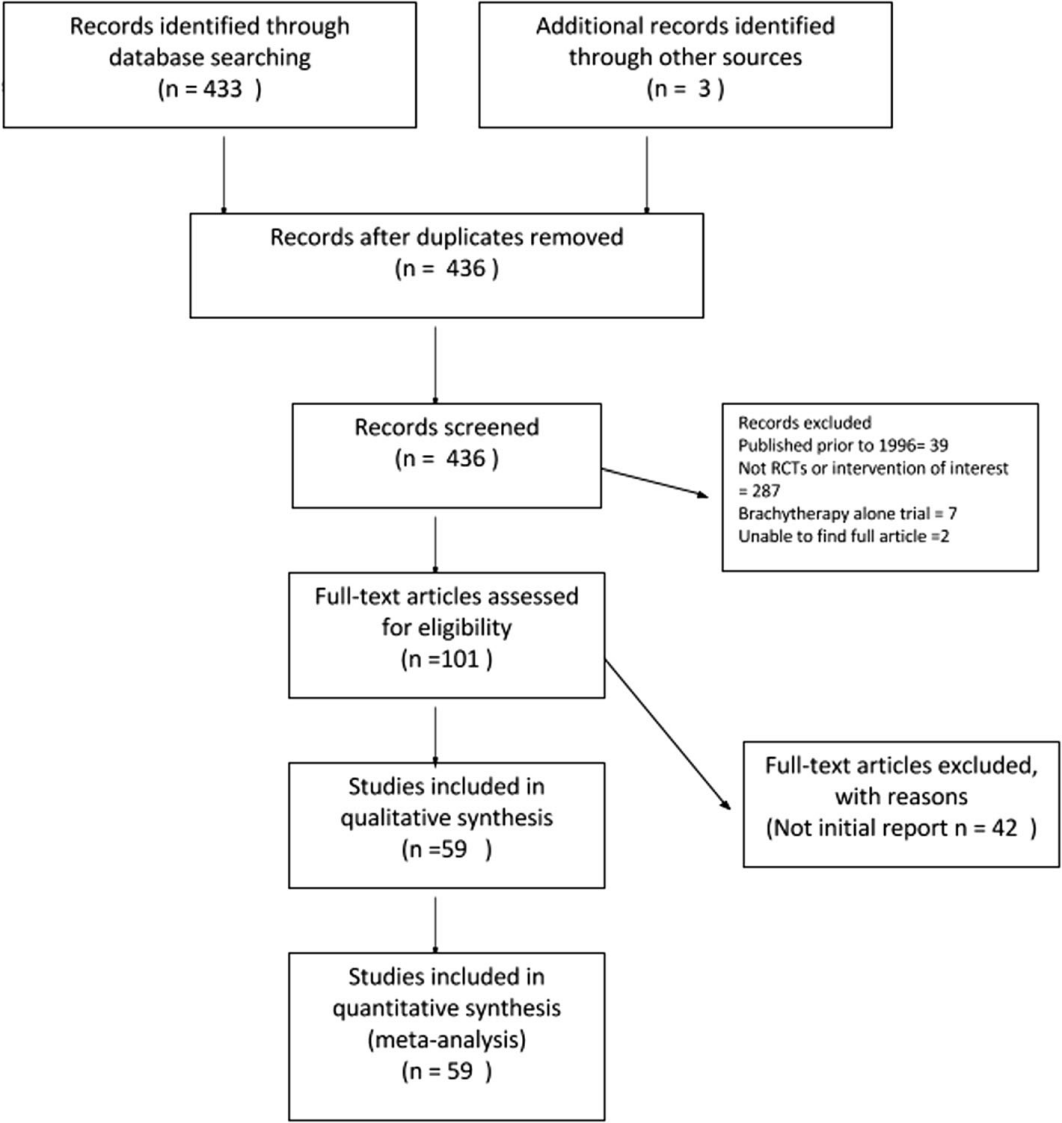
Results of the search strategy. Four hundred thirty-six records were identified. One hundred one full text articles were assessed for eligibility. Fifty-nine studies were included in the analysis

The result of the search strategy was summarized in Fig. 2. We identified 59 eligible trials.

#### Characteristics of trials

The characteristics of the trials are shown in Table 2. Majority of the trials treated patients with 3D conventional radiotherapy techniques. 73% of the trials had a sample size of more than 200 patients and 80% of trials were not industry sponsored.

**Table 2:** Characteristics of all trials. Majority of trials treated patients with 3D conventional techniques, had a sample size of 200 or more, were not industry sponsored, were listed in clinicaltrials.gov, were RT focused, had non-overall survival as the primary efficacy endpoint and were published in Europe. There were more trials published in radiotherapy journals, published form 1996-2005, published in journals with impact factor of more then 15. Majority of trials were non-cooperative group trials, did not publish their trial protocol, did not publish their QA processes, and did not have biases in reporting of primary efficacy endpoints and toxicity

Characteristics	N	%
RT Technique		
2D	1	2
3D Conventional	33	56
IMRT only	5	8
Multiple techniques	11	19
Not specified	9	15
Sample size		
≤ 200	16	27
> 200	43	73
Sponsorship		
Industry	12	20
Not industry	33	56
Not reported	14	24
Listed in clinicaltrials.gov		
Yes	15	25
No	44	75
Types of RT trial		
RT focused	40	68
Not RT focused	19	32
Primary efficacy endpoint		
Overall survival	8	14
Non-overall survival	42	86
Region		
North America	21	36
Europe	31	52
Asia	3	5
Australia/New Zealand	3	5
International	1	2
Radiotherapy Journal		
Yes	17	29
No	42	71
Year of publication		
1996–2005	36	61
2006–2016	23	39
Impact factor		
≤ 15	18	31
> 15	41	69
Cooperative group		
Yes	23	39
No	36	61
Trial protocol available		
Yes	14	24
No	45	76
QA process published		
Yes	16	27
No	43	83
Bias in reporting primary efficacy endpoint		
Yes	4	7
No	44	75
Not accessible	11	18
Bias in reporting toxicity		
Yes	13	22
No	46	78

### Quality of RT reporting

**Fig. 3:**
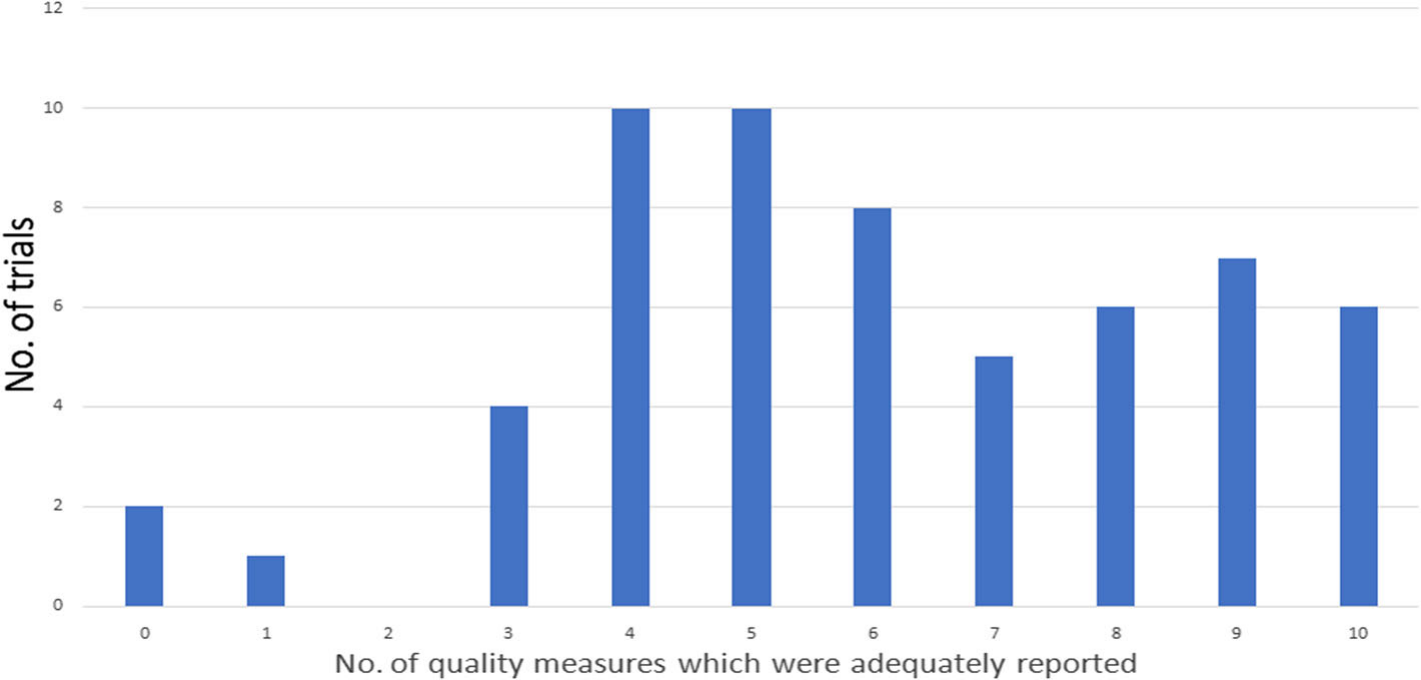
Bar graph showing number of quality measures which were adequately reported in all trials. X-axis shows number of quality measures reported. Y-axis shows number of trials reporting the corresponding number of quality measures adequately

Table 3 shows the number of trials and the quality measures they reported. Almost all trials reported target volume definition, total radiation dose and fractionation adequately. Radiation prescription point was reported in 37 (63%) trials. Description of dose planning procedures was present in 47 trials (80%). Verification procedures were reported in 27 trials (46%). OAR constraints, simulation procedures QA process for RT, QA process adherence reporting for RT was reported in approximately one third of all trials. Six trials (10%) reported all ten quality measures adequately. Figure 3 summarizes the number of quality measures that were adequately reported by the included trials. Trials included and their scores for each criteria is shown in Additional file 1.

**Table 3:** Quality of radiotherapy reporting. The quality measures are shown on the left column and the number and percentage of trials which reported that quality measure adequately shown on the right columns. This shows that the reporting of the quality measures were variable. Almost all trials reported target volume definition, radiation dose specification and fractionation specification adequately. Only one third of all trials reported OAR constraints, simulation procedures, and QA adherence reporting for RT adequately

Quality Measures	No. of trials which reported the quality measure adequately	% of trials which reported the quality measure adequately
Target volume definition	57	97
Radiation dose specification	58	99
Fractionation specification	57	98
Radiation prescription point specification	37	63
Description of Dose planning procedure	47	80
Organs at risk (OAR) constraints	20	34
Simulation procedures	21	36
Verification procedures	27	46
QA process for RT	24	41
QA process adherence reporting for RT	17	29

### Factors associated with adequate quality reporting

Twenty-four trials (40%) reported seven criteria or more adequately. Multivariate analysis showed that trials that published their QA results were 4 times more likely than trials that did not published their QA results {odds ratio (OR) 4.3, 95% confidence interval (CI) 0.99 to 19 and *p* value (*p*) = 0.05), and cooperative group trials were more 5 times more likely to have adequate quality in reporting (OR4.8, 95% CI 1.0 to 23.0, *p* = 0.047) compared to non-cooperative group trials. Other factors including region, primary outcome, industry sponsorship, trial design, sample size, journal’s impact factor, publication in radiotherapy focused journals, year of publication, listed in trial registry and trial protocol availability did not predict for adequate quality of reporting (Additional file 2).

### Impact on bias in reporting of primary efficacy and toxicity endpoints

Univariable logistic regression showed that trials with adequate quality of RT reporting were less likely to have bias in reporting of treatment toxicity. (OR 0.20. CI 0.04 to 0.99, *p* = 0.049).

## Discussion

To our knowledge, this is the first study to examine the quality of prostate radiotherapy reporting in RCTs of prostate cancer. Our study shows the quality of prostate radiotherapy reporting in RCTs of prostate cancer is variable, with only 40% of included trials reporting seven or more quality measures adequately. The reporting of target volumes, radiation dose and fractionation specifications were adequate in almost all trials. However, OAR constraints, simulation procedures QA process for RT, QA process adherence reporting for RT was poor and was only reported in approximately one third of all trials. Multivariate analysis of the trial and publication characteristics showed that cooperative group trials and trials which published their QA process and were more likely to be associated with adequate quality of radiotherapy reporting.

These findings emphasize the need to standardize the way we report radiotherapy treatment. Documenting and reporting the results of a QA process is an important component of radiotherapy in RCTs [10] Radiotherapy protocol deviations have been shown to lead to adverse outcomes for patients. Ohri et al. performed a meta-analysis to determine the impact of radiotherapy protocol deviations on clinical outcomes in cooperative group trials. He found that the frequency of RT QA deviations ranged from 8 to 71% in the included studies and this was associated with a significant decrease in OS (HR of death = 1.74, 95% confidence interval [CI] = 1.28 to 2.35; *P* < .001) [11]. This highlights the need for trials to adhere to protocol radiotherapy planning guidelines if patients are to benefit from the proposed treatment.

In Hodgkin’s lymphoma, Weiner et al. reported a randomized controlled trial of intensive chemotherapy with or without low dose radiation in the treatment of stage IIB to IV Hodgkin’s disease in paediatric patients [12]. An early report revealed no advantage of RT in this group of patients. However, in a subsequent evaluation of the data, it was found that there was a 10% survival advantage in patients receiving compliant RT. As much as 30% of patients had treatment deviation which included RT to involved sites. This highlights the need for studies to report protocol compliance. This may encourage and lead to improvements of processes to reduce protocol deviations.

The global quality assurance of Radiation Therapy Clinical Trials Harmonization group aims to broaden the acceptance of clinical trial results by harmonizing and improving the quality assurance of RT implemented world-wide. This will provide a framework for quality assurance in clinical trials [13].

The CONSORT statement proposes broad recommendations for reporting of interventions [14]. The updated CONSORT extension for non-pharmacologic treatment checklist entails reporting of precise details of both the experimental treatment and its comparator. However, specific recommendations are lacking for reporting of radiotherapy treatment. Bekelman and colleagues use six parameters, including target volume description, radiation dose specification, fractionation specification, radiation prescription point specification, QA process use and QA process adherence reporting to assess the quality of RT treatment reporting in Hodgkin’s and Non-Hodgkin’s Lymphoma trials. With improvement in technology, the way we treat patients with radiotherapy is changing. Computed tomography (CT) planning is currently the standard for treatment for many tumour subsites. CT planning allows us to visualize tumor volumes as well as organs at risk [15]. In addition to assessing tumour coverage, we are able to assess OAR constraints using dose volume histograms (DVH). There is increased emphasis on simulation and verification procedures with the implementation of image guided radiotherapy (IGRT) in the treatment of many tumour subsites [16]. In view of this, for our study we adopted 4 additional criteria from the recommendations by RTOG in addition to the 6 proposed by Bentzen et al. [17]and included description of simulation procedures, OAR constraints, simulation procedures and verification procedures in our assessment of quality of radiotherapy reporting.

Up to two thirds of trials reported prescription method adequately. The prescription method is integral to radiotherapy planning and delivery. With increasing use of IMRT for the treatment of prostate cancer, it is important for clinical trials to follow ICRU 83 in the prescribing and reporting of IMRT treatments [18].

Only one third of trials reported organs at risk (OAR) constraints adequately. OARs are extremely important in radiotherapy treatment planning as they typically represent avoidance structures that the planning system would minimize radiation dose delivery to. Increased doses to organs at risk will increase treatment toxicities. Not reporting organs at risk constraints will not allow the readers interpret the toxicity outcomes of the trials properly. Furthermore, overly conservative OAR constraints might lead to compromised target volume coverage, leading to increased risk of tumour recurrence.

One would expect the quality of radiotherapy reporting to improve over the years with increasing use of sophisticated technology for radiotherapy planning and delivery. We analyzed the year of publication as a variable to determine if trials published in the recent years were more likely to have adequate reporting. Interestingly, the quality of radiotherapy reporting did not improve over time. This may be because there are no published consensus guidelines on the reporting of RT treatment technique for trials including radiotherapy treatment.

We conducted a univariable logistic regression analysis, to determine if the quality of radiotherapy treatment reporting influenced the presence of bias in the reporting of primary efficacy and toxicity endpoints. We found that trials with adequate quality of RT reporting were less likely to have bias in reporting of treatment toxicity, but not primary efficacy endpoints. This is intuitive and suggests that trials with adequate reporting of RT treatment techniques may be more familiar with reporting standards of clinical trials.

The strengths of our study is that we used published tools to evaluate the quality of prostate RT reporting. In addition, we included only randomized trials for our study as the results from randomized trials are more likely to be impact clinical practice. Our study was limited by the relatively small sample size. In addition, not all the trial protocols were available for assessment. However, we felt that the primary trial report should describe at least seven key RT descriptors adequately as not all clinicians have time to examine the trial protocol in detail.

## Conclusion

The quality of prostate radiotherapy reporting in randomized controlled trials in prostate cancer is variable. Future efforts to develop consensus guidelines to standardize the reporting of radiotherapy treatment are warranted.

## Additional files

Additional file 1: Table of included studies and references. This table summarizes the characteristic of each study and the overall number of quality measures reported. The last column shows whether the study has reported seven or more quality measures adequately. 1 = Yes, 0 = No. (DOCX 48 kb)

Additional file 2: Univariate Analysis of Variables. This is a table showing the factors associated with adequate quality reporting in the univariate analysis. Cooperative group and availability of QA processes were significant and were included in the multivariable analysis (DOCX 19 kb)

## Abbreviations

2DRT: 2 dimensional RT
3DCRT: 3 dimensional conformal radiotherapy
CONSORT: Consolidated Standards of Reporting Trials
CT: Computed tomography
DVH: Dose volume histograms
EBRT: External beam radiotherapy
ICRU: International Commission on Radiation Units
IGRT: Image guided radiotherapy
IMRT: Intensity modulated radiotherapy
OAR: Organs at risk
OR: Odds Ratio
QA: Quality assurance
RCT: Randomized controlled trials
RT: Radiotherapy
RTOG: Radiotherapy Therapy Oncology Group

## References

[1] Burns PB, Rohrich RJ, Chung KC (2011). The levels of evidence and their role in evidenced based medicine. Plast Reconstr Surg.

[2] Chalmers TC, Smith H, Blackburn B (1981). A method for assessing the quality of a randomized control trial. Control Clin Trials.

[3] Albert JM, Das P. Quality indicators in radiation oncology. Int J Radiation Oncol Biol Phys. 2013;85(4):904e911. 10.1016/j.ijrobp.2012.08.038 23040217

[4] Begg C, Cho M, Eastwood S (1996). Improving the quality of reporting of randomized controlled trials: the CONSORT statement. JAMA.

[5] EQUATOR network. http://www.equator-network.org/reporting-guidelines/consort/. Accessed 20 Jul 2017.

[6] Globocan 2012: Estimate Cancer incidence, mortality and prevalence worldwide in 2012. http://globocan.iarc.fr/Default.aspx. Accessed 20 Jul 2017.

[7] Biagioli MC, Hoffe SE (2010). Emerging technologies in prostate cancer radiation therapy: improving the therapeutic window. Cancer Control.

[8] Bekelman JE, Yahalom J (2009). Quality of Radiotherapy Reporting in Randomized Controlled Trials of Hodgkin’s Lymphoma and Non-Hodgkin’s Lymphoma: A Systematic Review. Int J Radiat Oncol [Internet].

[9] Tan TH, Chen D, Soon YY, Tey JC (2016). Prevalence and predictors of bias in the reporting of primary efficacy and toxicity endpoints in randomized clinical trials of radiation oncology. J Med Imaging Radiat Oncol.

[10] Perez CA, Gardner P, Glasgow GP (1984). Radiotherapy quality assurance in clinical trials. Int J Radiat Oncol Biol Phys.

[11] Ohri N, Shen X, Dicker AP, Doyle LA, Harrison AS, Showalter TN (2013). Radiotherapy protocol deviations and clinical outcomes: a meta-analysis of cooperative group clinical trials. J Natl Cancer Inst [Internet].

[12] Weiner MA, Leventhal B, Brecher ML, Marcus RB, Cantor A, Gieser PW, Ternberg JL, Behm FG, Wharam MD Jr, Chauvenet AR. Randomized study of intensive MOPP-ABVD with or without low-dose total-nodal radiation therapy in the treatment of stages IIB, IIIA2, IIIB, and IV Hodgkin's disease in pediatric patients: a Pediatric Oncology Group study. J Clin Oncol. 1997;15(8):2769–79. 10.1200/JCO.1997.15.8.2769 9256118

[13] Global quality assurance of radiation therapy clinical trials harmonisation group. https://rtqaharmonization.com/. Accessed 3 June 2018.

[14] Moher D, Schulz KF, Altman DG (2001). The CONSORT statement: revised recommendations for improving the quality of reports of parallel-group randomised trials. Lancet.

[15] Bucci M, k, Bevan A, Roach M (2005). Advances in radiation therapy: conventional to 3D, to IMRT and to 4D, and Beyond. CA: A Cancer J for clinicians.

[16] Gupta T, Narayan CA (2012). Image-guided radiation therapy: Physician’s perspectives. J Med Phys.

[17] Bentzen SM (1998). Towards evidence based radiation oncology: improving the design, analysis, and reporting of clinical outcome studies in radiotherapy. Radiother Oncol.

[18] Prescribe, Recording, and Reporting Intensity Modulated Photon-Beam Therapy (IMRT). (ICRU report 83). Journal of the International Commission on Radiation Units and Measurements. 2010;10(1).

