# Supplementary material for: Quality of radiotherapy reporting in randomized controlled trials of prostate cancer
Source: Radiat Oncol. 2018 Jun 7;13:108. doi: 10.1186/s13014-018-1053-7 (PMC5992848; doi:10.1186/s13014-018-1053-7)
Supplement: Supplementary file 1 — Table of included studies and references. This table summarizes the characteristic of each study and the overall number of quality measures reported. The last column shows whether the study has reported seven or more quality measures adequately. 1 = Yes, 0 = No. (DOCX 48 kb) [file 13014_2018_1053_MOESM1_ESM.docx]

**Additional file 1: Table of included studies**

| Title | Target volume | Total radiation dose | Fractionation | Prescription point | Description of Dose planning procedure | OAR constraints | Simulation procedures | Verification procedures | QA process | Adherence to QA | No. of quality measures reported | Adequate quality |
| --- | --- | --- | --- | --- | --- | --- | --- | --- | --- | --- | --- | --- |
| Pilepich^1^ (2001) | 1 | 1 | 1 | 1 | 0 | 0 | 0 | 0 | 1 | 0 | 5 | 0 |
| Roach^2^ (2003) | 1 | 1 | 1 | 1 | 1 | 1 | 0 | 0 | 0 | 0 | 6 | 0 |
| Wiegel^3^ (2009) | 1 | 1 | 1 | 1 | 0 | 0 | 0 | 0 | 0 | 0 | 4 | 0 |
| Bolla^4^ (1997) | 1 | 1 | 1 | 1 | 0 | 0 | 0 | 0 | 1 | 0 | 5 | 0 |
| Wallner^5^ (2005) | 1 | 1 | 0 | 0 | 0 | 0 | 0 | 0 | 0 | 0 | 1 | 0 |
| Pollack^6^ (2000) | 1 | 1 | 1 | 1 | 1 | 1 | 0 | 0 | 0 | 0 | 6 | 0 |
| Zietman^7^ (2005) | 1 | 1 | 1 | 0 | 1 | 0 | 1 | 1 | 0 | 0 | 6 | 0 |
| Hoskin^8^ (2007) | 1 | 1 | 1 | 0 | 1 | 0 | 0 | 0 | 0 | 0 | 4 | 0 |
| Arcangeli^9^ (2010) | 1 | 1 | 1 | 0 | 1 | 0 | 1 | 1 | 0 | 0 | 6 | 0 |
| Yeoh^10^ (2003) | 1 | 1 | 1 | 1 | 1 | 0 | 0 | 0 | 0 | 0 | 5 | 0 |
| Widmark^11^ (2009) | 1 | 1 | 1 | 1 | 1 | 0 | 0 | 0 | 0 | 0 | 5 | 0 |
| Ward^12^ (2011) | 1 | 1 | 1 | 1 | 1 | 0 | 0 | 0 | 0 | 0 | 5 | 0 |
| Aluwini^13^ (2015) | 1 | 1 | 1 | 0 | 0 | 0 | 0 | 0 | 0 | 0 | 3 | 0 |
| Lennernas^14^ (2015) | 1 | 1 | 1 | 0 | 1 | 0 | 0 | 0 | 0 | 0 | 4 | 0 |
| Freytag^15^ (2014) | 0 | 1 | 1 | 0 | 1 | 0 | 0 | 0 | 0 | 0 | 3 | 0 |
| Creak^16^ (2013) | 1 | 1 | 1 | 1 | 1 | 0 | 0 | 1 | 0 | 0 | 6 | 0 |
| Heemsberg^17^ (2013) | 1 | 1 | 1 | 0 | 1 | 0 | 1 | 1 | 0 | 0 | 6 | 0 |
| Mottet^18^ (2012) | 1 | 1 | 1 | 0 | 1 | 0 | 0 | 0 | 1 | 0 | 5 | 0 |
| Donnelly^19^ (2010) | 1 | 1 | 1 | 0 | 1 | 0 | 0 | 0 | 0 | 0 | 4 | 0 |
| Hirano^20^ (2010) | 1 | 1 | 1 | 0 | 1 | 0 | 0 | 0 | 0 | 0 | 4 | 0 |
| Pommier^21^ (2007) | 1 | 1 | 1 | 0 | 1 | 0 | 0 | 0 | 0 | 0 | 4 | 0 |
| Chin^22^ (2008) | 1 | 1 | 1 | 0 | 1 | 0 | 0 | 0 | 0 | 0 | 4 | 0 |
| Akakura^23^ (2006) | 1 | 1 | 1 | 0 | 0 | 0 | 0 | 0 | 0 | 0 | 3 | 0 |
| Tyrrell^24^ (2005) | 0 | 0 | 0 | 0 | 0 | 0 | 0 | 0 | 0 | 0 | 0 | 0 |
| Sathya^25^ (2005) | 1 | 1 | 1 | 1 | 1 | 0 | 0 | 0 | 0 | 0 | 5 | 0 |
| Yamanaka^26^ (2005) | 1 | 1 | 1 | 0 | 0 | 0 | 0 | 1 | 0 | 0 | 4 | 0 |
| Windsor^27^ (2004) | 1 | 1 | 1 | 0 | 1 | 0 | 1 | 0 | 0 | 0 | 5 | 0 |
| Fransson^28^ (2001) | 1 | 1 | 1 | 0 | 1 | 0 | 0 | 0 | 0 | 0 | 4 | 0 |
| Van Cangh^29^ (1998) | 1 | 1 | 1 | 1 | 1 | 0 | 0 | 0 | 0 | 0 | 4 | 0 |
| Zietman^30^ (1996) | 1 | 1 | 1 | 0 | 0 | 0 | 0 | 0 | 0 | 0 | 3 | 0 |
| Beckendorf^31^ (2011) | 1 | 1 | 1 | 1 | 1 | 1 | 1 | 1 | 1 | 1 | 10 | 1 |
| Dearnaley^32^ (2012) | 1 | 1 | 1 | 1 | 1 | 1 | 1 | 1 | 1 | 1 | 10 | 1 |
| Zapatero^33^ (2015) | 1 | 1 | 1 | 1 | 1 | 1 | 0 | 1 | 0 | 0 | 7 | 1 |
| Norkus^34^ (2009) | 1 | 1 | 1 | 1 | 1 | 1 | 1 | 1 | 0 | 0 | 8 | 1 |
| Blanchard^35^ (2016) | 1 | 1 | 1 | 1 | 1 | 0 | 1 | 1 | 0 | 0 | 7 | 1 |
| Hoffman^36^ (2014) | 1 | 1 | 1 | 1 | 1 | 1 | 0 | 1 | 0 | 0 | 7 | 1 |
| Pollack^37^ (2013) | 1 | 1 | 1 | 1 | 1 | 1 | 0 | 1 | 1 | 1 | 9 | 1 |
| Norkus^38^ (2013) | 1 | 1 | 1 | 0 | 1 | 1 | 1 | 1 | 0 | 0 | 7 | 1 |
| Vainshtein^39^ (2012) | 1 | 1 | 1 | 1 | 1 | 1 | 1 | 1 | 0 | 0 | 8 | 1 |
| Jones^40^ (2011) | 1 | 1 | 1 | 1 | 1 | 1 | 0 | 1 | 1 | 1 | 9 | 1 |
| Armstrong^41^ (2011) | 1 | 1 | 1 | 1 | 1 | 1 | 1 | 0 | 0 | 0 | 8 | 1 |
| Lukka^42^ (2005) | 1 | 1 | 1 | 1 | 1 | 0 | 1 | 1 | 1 | 0 | 8 | 1 |
| Koper^43^ (1999) | 1 | 1 | 1 | 1 | 1 | 0 | 1 | 1 | 0 | 0 | 7 | 1 |
| Lawton^44^ (2001) | 1 | 1 | 1 | 1 | 0 | 0 | 0 | 0 | 1 | 0 | 5 | 0 |
| Dearnaley^45^ (2007) | 1 | 1 | 1 | 1 | 1 | 0 | 0 | 0 | 1 | 0 | 6 | 0 |
| Thompson^46^ (2006) | 1 | 1 | 1 | 0 | 0 | 0 | 0 | 0 | 1 | 1 | 5 | 0 |
| D'Amico^47^ (2004) | 1 | 1 | 1 | 1 | 1 | 0 | 0 | 0 | 1 | 1 | 6 | 0 |
| James^48^ (2016) | 1 | 1 | 1 | 0 | 1 | 0 | 0 | 0 | 1 | 1 | 0 | 0 |
| Denham^49^ (2011) | 1 | 1 | 1 | 1 | 1 | 1 | 0 | 1 | 1 | 1 | 9 | 1 |
| Denham^50^ (2014) | 1 | 1 | 1 | 1 | 1 | 1 | 1 | 1 | 1 | 1 | 10 | 1 |
| Peeters^51^ (2006) | 1 | 1 | 1 | 1 | 1 | 1 | 1 | 0 | 1 | 1 | 9 | 1 |
| Michalski^52^ (2013) | 1 | 1 | 1 | 1 | 1 | 1 | 1 | 1 | 1 | 1 | 10 | 1 |
| Bolla^53^ (2005) | 1 | 1 | 1 | 1 | 1 | 0 | 1 | 1 | 1 | 1 | 9 | 1 |
| Hanks^54^ (2003) | 1 | 1 | 1 | 1 | 0 | 1 | 0 | 1 | 1 | 1 | 8 | 1 |
| Ghadjar^55^ (2015) | 1 | 1 | 1 | 1 | 1 | 1 | 1 | 1 | 1 | 1 | 10 | 1 |
| Pisansky^56^ (2015) | 1 | 1 | 1 | 1 | 1 | 1 | 1 | 1 | 1 | 1 | 10 | 1 |
| Bolla^57^ (2009) | 1 | 1 | 1 | 1 | 1 | 0 | 1 | 1 | 1 | 1 | 9 | 1 |
| Dearnaley^58^ (1999) | 1 | 1 | 1 | 1 | 1 | 0 | 0 | 1 | 1 | 1 | 8 | 1 |
| Hamdy^59^ (2016) | 1 | 1 | 1 | 1 | 1 | 1 | 1 | 1 | 1 | 0 | 9 | 1 |

Legend: 1- yes, 0- No

**References**

1. Pilepich MV, Winter K, John MJ et al. Phase III radiation therapy oncology group (RTOG) trial 86-10 of androgen deprivation adjuvant to definitive radiotherapy in locally advanced carcinoma of the prostate. Int J Radiat Oncol Biol Phys. 2001 Aug 1;50(5):1243-52.

2. Roach M 3rd, DeSilvio M, Lawton C et al. Phase III trial comparing whole-pelvic versus prostate-only radiotherapy and neoadjuvant versus adjuvant combined androgen suppression: Radiation Therapy Oncology Group 9413. J Clin Oncol. 2003 May 15;21(10):1904-11.

3. Wiegel T, Bottke D, Steiner U et al. Phase III postoperative adjuvant radiotherapy after radical prostatectomy compared with radical prostatectomy alone in pT3 prostate cancer with postoperative undetectable prostate-specific antigen: ARO 96-02/AUO AP 09/95. J Clin Oncol. 2009 Jun 20;27(18):2924-30.

# 4. Bolla M, Gonzalez D, Ward P et al. Improved survival in patients with locally advanced prostate cancer treated with radiotherapy and goserelin. [N Engl J Med.](https://www.ncbi.nlm.nih.gov/pubmed/9233866) 1997 Jul 31;337(5):295-300.

5. Wallner K, Merrick G, True L et al. 20 Gy versus 44 Gy supplemental beam radiation with Pd-103 prostate brachytherapy: preliminary biochemical outcomes from a prospective randomized multi-center trial. Radiother Oncol. 2005 Jun;75(3):307-10.

6. Pollack A, Zagars GK, Smith LG et al. Preliminary results of a randomized radiotherapy dose-escalation study comparing 70 Gy with 78 Gy for prostate cancer. J Clin Oncol. 2000 Dec 1;18(23):3904-11.

7. Zietman AL, DeSilvio ML, Slater JD et al. Comparison of conventional-dose vs high-dose conformal radiation therapy in clinically localized adenocarcinoma of the prostate: a randomized controlled trial. JAMA. 2005 Sep 14;294(10):1233-9.

8. Hoskin PJ, Motohashi K, Bownes P et al. High dose rate brachytherapy in combination with external beam radiotherapy in the radical treatment of prostate cancer: initial results of a randomised phase three trial. Radiother Oncol. 2007 Aug;84(2):114-20.

9. Arcangeli G, Saracino B, Gomellini S et al. A prospective phase III randomized trial of hypofractionation versus conventional fractionation in patients with high-risk prostate cancer. Int J Radiat Oncol Biol Phys. 2010 Sep 1;78(1):11-8.

10. Yeoh EE, Fraser RJ, McGowan RE et al. Evidence for efficacy without increased toxicity of hypofractionated radiotherapy for prostate carcinoma: early results of a Phase III randomized trial. Int J Radiat Oncol Biol Phys. 2003 Mar 15;55(4):943-55.

11. Widmark A1, Klepp O, Solberg A et al. Endocrine treatment, with or without radiotherapy, in locally advanced prostate cancer (SPCG-7/SFUO-3): an open randomised phase III trial. Lancet. 2009 Jan 24;373(9660):301-8.

12. Ward P, Mason M, Ding K et al. Impact of Radiotherapy When Added to Androgen-Deprivation Therapy for Locally Advanced Prostate Cancer: Lancet. 2011. Dec 17;378(9809):2104-11.

13. Aluwini S, Pos F, Shimmel E et al. Hypofractionated versus conventionally fractionated radiotherapy for patients with prostate cancer (HYPRO): Final results results from a randomised non-inferiority phase 3 trial. Lancet. 2015. Mar;16(3):274-83

14. LennernÃ¤s B, Majumder K, Damber JE et al. Radical prostatectomy versus high-dose irradiation in localized/locally advanced prostate cancer: A Swedish multicenter randomized trial with patient-reported outcomes. Acta Oncol. 2015 Jun;54(6):875-81.

15. Freytag SO, Stricker H, Lu M, Elshaikh M et al. Prospective randomized phase 2 trial of intensity modulated radiation therapy with or without oncolytic adenovirus-mediated cytotoxic gene therapy in intermediate-risk prostate cancer. Int J Radiat Oncol Biol Phys. 2014 Jun 1;89(2):268-76.

16. Creak A, Hall E, Horwich A et al. Randomised pilot study of dose escalation using conformal radiotherapy in prostate cancer: long-term follow-up. Br J Cancer. 2013 Aug 6;109(3):651-7.

17. Heemsbergen WD, Al-Mamgani A, Witte MG et al. Radiotherapy with rectangular fields is associated with fewer clinical failures than conformal fields in the high-risk prostate cancer subgroup: results from a randomized trial. Radiother Oncol. 2013 May;107(2):134-9.

18. Mottet N, Peneau M, Mazeron JJ et al. Addition of radiotherapy to long-term androgen deprivation in locally advanced prostate cancer: an open randomised phase 3 trial. Eur Urol. 2012 Aug;62(2):213-9.

19. Donnelly BJ, Saliken JC, Brasher PM et al. A randomized trial of external beam radiotherapy versus cryoablation in patients with localized prostate cancer. Cancer. 2010 Jan 15;116(2):323-30.

20. Hirano D, Nagane Y, Satoh K et al. Neoadjuvant LHRH analog plus estramustine phosphate combined with three-dimensional conformal radiotherapy for intermediate- to high-risk prostate cancer: a randomized study. Int Urol Nephrol. 2010 Mar;42(1):81-8.

21. Pommier P, Chabaud S, Lagrange JL et al. Is there a role for pelvic irradiation in localized prostate adenocarcinoma? Preliminary results of GETUG-01. J Clin Oncol. 2007 Dec 1;25(34):5366-73.

22. Chin JL, Ng CK, Touma NJ et al. Randomized trial comparing cryoablation and external beam radiotherapy for T2C-T3B prostate cancer. Prostate Cancer Prostatic Dis. 2008;11(1):40-5.

23. Akakura K, Suzuki H, Fujimoto et al. A randomized trial comparing radical prostatectomy plus endocrine therapy versus external beam radiotherapy plus endocrine therapy for locally advanced prostate cancer: results at median follow-up of 102 months. [Jpn J Clin Oncol.](https://www.ncbi.nlm.nih.gov/pubmed/?term=A+randomized+trial+comparing+radical+prostatectomy+plus+endocrine+therapy+versus+external+beam+radiotherapy+plus+endocrine+therapy+for+locally+advanced+prostate+cancer%3A+results+at+median+follow-up+of+102+months.) 2006 Dec;36(12):789-93

24. Tyrrell CJ, Payne H, See WA et al. Bicalutamide ('Casodex') 150 mg as adjuvant to radiotherapy in patients with localised or locally advanced prostate cancer: results from the randomised Early Prostate Cancer Programme. Radiother Oncol. 2005 Jul;76(1):4-10.

25. Sathya JR, Davis IR, Julian JA et al. Randomized trial comparing iridium implant plus external-beam radiation therapy with external-beam radiation therapy alone in node-negative locally advanced cancer of the prostate. Prostate. 2005 Apr 1;63(1):56-64.

26. Yamanaka H, Ito K, Naito S et al. Effectiveness of adjuvant intermittent endocrine therapy following neoadjuvant endocrine therapy and external beam radiation therapy in men with locally advanced prostate cancer. Prostate. 2005 Apr 1;63(1):56-64.

27. Windsor PM, Nicol KF, Potter J. et al. A randomized, controlled trial of aerobic exercise for treatment-related fatigue in men receiving radical external beam radiotherapy for localized prostate carcinoma. Cancer. 2004 Aug 1;101(3):550-7.

28. Fransson P, Damber JE, Tomic R et al. Quality of life and symptoms in a randomized trial of radiotherapy versus deferred treatment of localized prostate carcinoma. Cancer. 2001 Dec 15;92(12):3111-9.

29. Van Cangh PJ, Richard F, Lorge F et al. Adjuvant radiation therapy does not cause urinary incontinence after radical prostatectomy: results of a prospective randomized study. J Urol. 1998 Jan;159(1):164-6.

30. Zietman AL, Dallow KC, McManus PA et al. Time to second prostate-specific antigen failure is a surrogate endpoint for prostate cancer death in a prospective trial of therapy for localized disease. Urology. 1996 Feb;47(2):236-9.

31. Beckendorf V, Guerif S, Le Prise E et al. 70 Gy versus 80 Gy in localized prostate cancer: 5-year results of GETUG 06 randomized trial. Int J Radiat Oncol Biol Phys. 2011 Jul 15;80(4):1056-63.

32. [Dearnaley D](https://www.ncbi.nlm.nih.gov/pubmed/?term=Dearnaley%20D%5BAuthor%5D&cauthor=true&cauthor_uid=22169269), [Syndikus I](https://www.ncbi.nlm.nih.gov/pubmed/?term=Syndikus%20I%5BAuthor%5D&cauthor=true&cauthor_uid=22169269), [Sumo G](https://www.ncbi.nlm.nih.gov/pubmed/?term=Sumo%20G%5BAuthor%5D&cauthor=true&cauthor_uid=22169269) et al. Conventional versus hypofractionated high-dose intensity-modulated radiotherapy for prostate cancer: preliminary safety results from the CHHiP randomised controlled trial. Lancet Oncol. 2012 Jan;13(1):43-54.

33. Zapatero A, Guerrero A, Maldonado X et al. High-dose radiotherapy with short-term or long-term androgen deprivation in localised prostate cancer (DART01/05 GICOR): a randomised, controlled, phase 3 trial. Lancet Oncol. 2015 Mar;16(3):320-7.

34. Norkus D, Miller A, Kurtinaitis J et al. A randomized trial comparing hypofractionated and conventionally fractionated three-dimensional external-beam radiotherapy for localized prostate adenocarcinoma : a report on acute toxicity. Strahlenther Onkol. 2009 Nov;185(11):715-21.

35. Blanchard P, Faivre L, Lesaunier F et al. Outcome According to Elective Pelvic Radiation Therapy in Patients With High-Risk Localized Prostate Cancer: A Secondary Analysis of the GETUG 12 Phase 3 Randomized Trial. Int J Radiat Oncol Biol Phys. 2016 Jan 1;94(1):85-92.

36. Hoffman KE, Voong KR, Pugh TJ et al. Risk of late toxicity in men receiving dose-escalated hypofractionated intensity modulated prostate radiation therapy: results from a randomized trial. Int J Radiat Oncol Biol Phys. 2014 Apr 1;88(5):1074-84.

37. Pollack A, Walker G, Horwitz EM et al. Randomized trial of hypofractionated external-beam radiotherapy for prostate cancer. J Clin Oncol. 2013 Nov 1;31(31):3860-8.

38. Norkus D, Karklelyte A, Engels B et al. A randomized hypofractionation dose escalation trial for high risk prostate cancer patients: interim analysis of acute toxicity and quality of life in 124 patients. Radiat Oncol. 2013 Sep 4;8:206.

39. Vainshtein J, Abu-Isa E, Olson KB et al. Randomized phase II trial of urethral sparing intensity modulated radiation therapy in low-risk prostate cancer: implications for focal therapy. Radiat Oncol. 2012 Jun 9;7:82.

40. Jones CU, Hunt D, McGowan DG et al. Radiotherapy and short-term androgen deprivation for localized prostate cancer. N Engl J Med. 2011 Jul 14;365(2):107-18.

41. Armstrong JG, Gillham CM, Dunne MT et al. A randomized trial (Irish clinical oncology research group 97-01) comparing short versus protracted neoadjuvant hormonal therapy before radiotherapy for localized prostate cancer. Int J Radiat Oncol Biol Phys. 2011 Sep 1;81(1):35-45.

42. Lukka H, Hayter C, Julian JA et al. Randomized trial comparing two fractionation schedules for patients with localized prostate cancer. J Clin Oncol. 2005 Sep 1;23(25):6132-8.

43. Koper PC, Stroom JC, van Putten WL et al. Acute morbidity reduction using 3DCRT for prostate carcinoma: a randomized study. Int J Radiat Oncol Biol Phys. 1999 Mar 1;43(4):727-34.

44. Lawton CA, Winter K, Murray K et al. Updated results of the phase III Radiation Therapy Oncology Group (RTOG) trial 85-31 evaluating the potential benefit of androgen suppression following standard radiation therapy for unfavorable prognosis carcinoma of the prostate. Int J Radiat Oncol Biol Phys. 2001 Mar 15;49(4):937-46.

45. Dearnaley DP, Sydes MR, Graham JD et al. Escalated-dose versus standard-dose conformal radiotherapy in prostate cancer: first results from the MRC RT01 randomised controlled trial. Lancet Oncol. 2007 Jun;8(6):475-87.

46. Thompson IM Jr, Tangen CM, Paradelo J et al. Adjuvant radiotherapy for pathologically advanced prostate cancer: a randomized clinical trial. JAMA. 2006 Nov 15;296(19):2329-35.

47. D'Amico AV, Manola J, Loffredo M et al. 6-month androgen suppression plus radiation therapy vs radiation therapy alone for patients with clinically localized prostate cancer: a randomized controlled trial. JAMA. 2004 Aug 18;292(7):821-7.

48. James ND, Spears MR, Clarke NW et al. Failure-Free Survival and Radiotherapy in Patients With Newly Diagnosed Nonmetastatic Prostate Cancer: Data From Patients in the Control Arm of the STAMPEDE Trial. JAMA Oncol. 2016 Mar;2(3):348-57.

49. Denham JW, Steigler A, Lamb DS, et al. Short-term neoadjuvant androgen deprivation and radiotherapy for locally advanced prostate cancer: 10-year data from the TROG 96.01 randomised trial. Lancet Oncol. 2011 May;12(5):451-9.

50. Denham JW, Joseph D, Lamb DS et al. Short-term androgen suppression and radiotherapy versus intermediate-term androgen suppression and radiotherapy, with or without zoledronic acid, in men with locally advanced prostate cancer (TROG 03.04 RADAR): an open-label, randomised, phase 3 factorial trial. Lancet Oncol. 2014 Sep;15(10):1076-89.

51. Peeters ST, Heemsbergen WD, Koper PC et al. Dose-response in radiotherapy for localized prostate cancer: results of the Dutch multicenter randomized phase III trial comparing 68 Gy of radiotherapy with 78 Gy. J Clin Oncol. 2006 May 1;24(13):1990-6.

52. Michalski JM, Yan Y, Watkins-Bruner D et al. Preliminary toxicity analysis of 3-dimensional conformal radiation therapy versus intensity modulated radiation therapy on the high-dose arm of the Radiation Therapy Oncology Group 0126 prostate cancer trial. Int J Radiat Oncol Biol Phys. 2013 Dec 1;87(5):932-8.

53. Bolla M, Van Poppel H, Collette L et al. Preliminary results for EORTC trial 22911: radical prostatectomy followed by postoperative radiotherapy in prostate cancers with a high risk of progression. [Lancet.](https://www.ncbi.nlm.nih.gov/pubmed/16099293) 2005 Aug 13-19;366(9485):572-8.

54. Hanks GE, Pajak TF, Porter A. Phase III trial of long-term adjuvant androgen deprivation after neoadjuvant hormonal cytoreduction and radiotherapy in locally advanced carcinoma of the prostate: the Radiation Therapy Oncology Group Protocol 92-02. [J Clin Oncol.](https://www.ncbi.nlm.nih.gov/pubmed/?term=Phase+III+trial+of+long-term+adjuvant+androgen+deprivation+after+neoadjuvant+hormonal+cytoreduction+and+radiotherapy+in+locally+advanced+carcinoma+of+the+prostate%3A+the+Radiation+Therapy+Oncology+Group+Protocol+92-02.) 2003 Nov 1;21(21):3972-8.

55. Ghadjar P, Hayoz S, Bernhard J et al. Acute Toxicity and Quality of Life After Dose-Intensified Salvage Radiation Therapy for Biochemically Recurrent Prostate Cancer After Prostatectomy: First Results of the Randomized Trial SAKK 09/10. J Clin Oncol. 2015 Dec 10;33(35):4158-66.

56. Pisansky TM, Hunt D, Gomella LG et al. Duration of androgen suppression before radiotherapy for localized prostate cancer: radiation therapy oncology group randomized clinical trial 9910. J Clin Oncol. 2015 Feb 1;33(4):332-9.

57. Bolla M, de Reijke TM, Van Tienhoven G et al. Duration of androgen suppression in the treatment of prostate cancer. N Engl J Med. 2009 Jun 11;360(24):2516-27.

58. Dearnaley DP, Khoo VS, Norman AR et al. Comparison of radiation side-effects of conformal and conventional radiotherapy in prostate cancer: a randomised trial. Lancet. 1999 Jan 23;353(9149):267-72.

59. Hamdy FC, Donovan JL, Lane JA et al. 10-Year Outcomes after Monitoring, Surgery, or Radiotherapy for Localized Prostate Cancer. N Engl J Med. 2016 Oct 13;375(15):1415-1424.
