# Supplementary material for: Quality of radiotherapy reporting in randomized controlled trials of prostate cancer
Source: Radiat Oncol. 2018 Jun 7;13:108. doi: 10.1186/s13014-018-1053-7 (PMC5992848; doi:10.1186/s13014-018-1053-7)
Supplement: Supplementary file 2 — Univariate Analysis of Variables. This is a table showing the factors associated with adequate quality reporting in the univariate analysis. Cooperative group and availability of QA processes were significant and were included in the multivariable analysis (DOCX 19 kb) [file 13014_2018_1053_MOESM2_ESM.docx]

**Additional file 1: Unvariate Analysis of variables**

| Factors associated with adequate quality reporting | | Odds ratio | 95% CI | P value |
| --- | --- | --- | --- | --- |
| Year of publication | 1996 to 2005 | Reference |  |  |
|  | 2006 to 2016 | 2.83 | 0.91 to 8.83 | 0.07 |
| Cooperative group | No | Reference |  |  |
|  | Yes | 2.96 | 1.00 to 8.77 | **0.05** |
| Region | North America | Reference |  |  |
|  | Others | 1.18 | 0.40 to 3.52 | 0.76 |
| Primary outcome | Overall survival | Reference |  |  |
|  | Others | 1.17 | 0.25 to 5.42 | 0.84 |
| Industry sponsored | Yes | Reference |  |  |
|  | No or not reported | 1.84 | 0.48 to 7.10 | 0.36 |
| Sample size | ≤200 | Reference |  |  |
|  | >200 | 2.67 | 0.91 to 7.86 | 0.075 |
| Published in radiotherapy focused journal | No | Reference |  |  |
|  | Yes | 1.19 | 0.37 to 3.80 | 0.77 |
| Trial design | Non-Radiotherapy focused | Reference |  |  |
|  | Radiotherapy focused | 0.917 | 0.30 to 2.78 | 0.88 |
| Listed in trial registry | Yes | Reference |  |  |
|  | No | 0.22 | 0.18 to 2.63 | 0.23 |
| Impact factor | ≤15 | Reference |  |  |
|  | >15 | 1.79 | 0.58 to 5.54 | 0.315 |
| Trial protocol available | Yes | Reference |  |  |
|  | No | 2.23 | 0.69 to 7.93 | 0.18 |
| QA process available | Yes | Reference |  |  |
|  | No | 4.9 | 1.42 to 17.0 | **0.01** |
